# Global burden of inflammatory bowel disease in children and adolescents, 1990–2021: trends, age-specific patterns and future projections

**DOI:** 10.3389/fped.2025.1670440

**Published:** 2025-09-03

**Authors:** Hai-Qing Tan, Qian-Kun Li, Mu-Rong Jiang, Dong-Hua Bin

**Affiliations:** The First Hospital of Hunan University of Chinese Medicine, Changsha, China

**Keywords:** inflammatory bowel disease, children and adolescents, global disease burden, very early-onset IBD, projections

## Abstract

**Introduction:**

Recent epidemiological trends have revealed a marked increase in incidence among children and adolescents. This study aimed to analyze the global burden of inflammatory bowel disease (IBD) among children and adolescents aged 0–19 years from 1990 to 2021 systematically, focusing on regional and age-specific trends and future projections, with the aim of informing global prevention and control strategies.

**Methods:**

Data on IBD incidence, mortality, and disability-adjusted life years (DALYs) were obtained for 204 countries and territories from the Global Burden of Disease Study 2021 (GBD 2021). Temporal trends were evaluated via the estimated annual percentage change (EAPC) and average annual percentage change (AAPC). A Bayesian age-period-cohort (BAPC) model was used to project the disease burden through 2050. The relationship between the sociodemographic index (SDI) and disease burden was also assessed, with Spearman's rank correlation coefficient applied for correlation analysis.

**Results:**

Globally, the incidence of IBD among children and adolescents remained stable (EAPC = −0.03%, 95% CI: −0.44–0.38), although marked regional disparities were observed. The incidence rates were stable or declined in high-income countries (e.g., high-income North America: EAPC = −1.07, 95% CI: −1.82–−0.32), whereas industrializing regions showed significant increases (e.g., East Asia: EAPC = 2.01, 95% CI: 1.02–3.19). Although very early-onset IBD (VEO-IBD) is rare, it is associated with disproportionately high mortality and DALY rates based on indirect estimates derived from the <5-year-old group, which may have inherent limitations. Globally, mortality has decreased by 51.6% and DALYs by 49.5%, yet the burden remains high in low-SDI regions. Projections suggest that by 2050, the incidence may reach 0.71 per 100,000, while mortality and DALY rates will continue to decline.

**Discussion:**

The global burden of IBD in children and adolescents is characterized by significant regional disparities, with VEO-IBD presenting unique challenges. Targeted interventions—including early diagnosis, enhanced multidisciplinary care, and international collaboration—are urgently needed, especially in low- and middle-income countries where resource allocation and disease management remain limited.

## 1 Introduction

Inflammatory bowel disease (IBD) is a chronic, nonspecific inflammatory condition of the gastrointestinal tract that primarily includes Crohn's disease (CD), ulcerative colitis (UC), and IBD-unclassified (IBD-U). It is characterized by an unknown etiology, a prolonged and unpredictable disease course, and recurrent episodes of relapse and remission (1, 2). The chronic, relapsing nature of IBD not only imposes a considerable economic burden on healthcare systems but also negatively impacts patients' psychological well-being, quality of life, and social functioning (3). These realities underscore the urgent need for continued research to inform future strategies for the prevention and clinical management of IBD.

Although IBD has traditionally been regarded as a disease of adulthood (4), recent epidemiological trends reveal a notable increase in incidence among children and adolescents. In regions such as Denmark and Canada, the annual growth rates have exceeded 8% (5). Pediatric patients face distinct challenges: approximately 35% experience micronutrient deficiencies, 25% suffer from growth retardation, and chronic inflammation may increase the risk of colorectal cancer later in life (6). Moreover, studies indicate that children diagnosed with IBD have a 50% increased risk of developing depression (7).

Particularly notable is the subgroup of very early-onset IBD (VEO-IBD), defined as onset before the age of six, which has attracted growing attention owning to its distinct genetic underpinnings and aggressive clinical course. Evidence indicates that patients with VEO-IBD often exhibit more severe disease manifestations compared to those with adult-onset IBD. This may be associated with differences in anatomical distribution (8). Furthermore, monogenic mutations are more frequently implicated in the pathogenesis of VEO-IBD than in later-onset forms (9).

Against this backdrop, the present study analyzes the global burden of IBD in individuals aged 0–19 years from 1990 to 2021, focusing on regional and age-specific patterns. The ≤19-year age range was selected because it encompasses key developmental stages in childhood and adolescence, includes major pediatric IBD subtypes, and aligns with the World Health Organization's definition of children and adolescents (10). This study has important implications for both prevention and clinical practice. From a prevention perspective, identifying regional patterns and associated risk factors can help guide targeted strategies. For example, industrializing countries with rising incidence rates may benefit from enhanced monitoring and intervention efforts aimed at mitigating environmental exposures. From a clinical standpoint, recognizing disparities in disease burden across age groups and regions can support more effective allocation of healthcare resources. Furthermore, to address the challenges of VEO-IBD, this study advocates the integration of genetic testing into clinical protocols to enable precision treatment and improve diagnostic accuracy. Although the GBD database does not directly distinguish the <6-year age group, thereby limiting data granularity, our use of the <5-year group provides a preliminary foundation for developing targeted surveillance systems and early interventions for high-risk subpopulations.

In summary, this study is critically important for improving global strategies for the prevention and management of pediatric and adolescent IBD.

## 2 Methods

### 2.1 Data sources and processing

This study utilized data from the Global Burden of Disease Study 2021 (GBD 2021), extracting epidemiological indicators of IBD among individuals aged 0–19 years across 204 countries and territories from 1990 to 2021. The indicators included incidence, disability-adjusted life years (DALYs), and mortality. The GBD integrates census data, disease registries, healthcare utilization records, and published literature, employing standardized methods to ensure cross-regional comparability (11, 12). Data were accessed through the Global Health Data Exchange (GHDx) platform (https://ghdx.healthdata.org/gbd-2021, accessed 30 Mar 2025), which is maintained by the Institute for Health Metrics and Evaluation (IHME). All the data were stratified by country/region, age group, and sex and processed via standard protocols for inclusion, data cleaning, missing value imputation, and bias adjustment to ensure comparability across locations and time periods. Both annual case counts and rates of IBD incidence, DALYs, and mortality were extracted, along with corresponding 95% uncertainty intervals (UIs) to enhance reliability and facilitate significance testing (13). UIs presented alongside estimates or projections indicate the range within which the true value of the disease burden is most likely to fall, reflecting variability and uncertainty in data sources, measurement error, and model assumptions. For instance, a 95% UI means there is a 95% probability that the actual value lies within this range. Wider UIs denote greater uncertainty and warrant more cautious interpretation of the findings (14).

### 2.2 Disease and population definitions

Following the GBD cause hierarchy from IHME, IBD was defined according to the International Classification of Diseases, 10th Revision (ICD-10), codes K50 and K51 (15). These codes encompass all the anatomical and clinical manifestations of IBD, without further classification by disease type or site. The pediatric and adolescent population was defined as individuals aged ≤19 years and further stratified into four age groups: <5 years, 5–9 years, 10–14 years, and 15–19 years. Very early-onset IBD (VEO-IBD) was defined as IBD diagnosed before the age of six (<5 years was chosen due to the age stratification used in the GBD database, which does not include a <6-year-old category).

### 2.3 Sociodemographic index (SDI)

The SDI is a composite metric that reflects a country's or region's level of socioeconomic development, incorporating data on income, education, and fertility rates. Analyzing disease burden in relation to the SDI allows for the assessment of how socioeconomic factors affect health outcomes and provides a framework for forecasting the public health impact of future economic changes (16). In this study, frontier analysis was also employed to evaluate the relative efficiency of countries and regions in achieving health outcomes, with a particular emphasis on the relationship between SDI and disease burden. This approach enables the quantification of performance gaps across various development levels (17). To examine the association between the SDI and IBD indicators (incidence, mortality, and DALYs), Spearman's rank correlation coefficient was employed. This nonparametric approach was selected to account for potential deviations from normality and non-linear relationships in the data, offering a robust measure of monotonic association (18).

### 2.4 Statistical analysis

Joinpoint regression was used to calculate the annual percentage change (APC) and its 95% confidence interval (CI), identifying statistically significant shifts in temporal trends. Joinpoint models segment the overall trend into discrete intervals on the basis of observed inflection points, allowing for the independent assessment of subperiod trends (19). The estimated annual percentage change (EAPC), derived from a log-linear regression model of the rate, was used to quantify temporal changes over the entire study period. The 95% CI of the EAPC was interpreted as follows: a CI entirely above 0 indicates an increasing trend; a CI entirely below 0 indicates a decreasing trend; and a CI including 0 indicates a stable trend (20). To evaluate the robustness of using the <5-year-old age group as a proxy for VEO-IBD, we performed a sensitivity analysis comparing temporal trends and disease burden estimates across alternative pediatric age groups available in the GBD database. Specifically, we examined incidence, mortality, and DALY rates in the 2–4-year, <5-year, and 5–9-year groups over the study period (1990–2021). This analysis aimed to assess the consistency of observed trends and determine the potential influence of age group definitions on our key conclusions regarding the VEO-IBD burden (21).

### 2.5 Projection

A Bayesian age–period–cohort (BAPC) model was used to project IBD incidence, mortality, and DALY rates in children and adolescents from 2022 to 2050. Based on Bayesian statistical principles, the model incorporates age, period, and cohort effects to estimate long-term disease trends. It assumes that future trends will resemble historical patterns and that these effects are independent. The accuracy of the projections depends on the quality and completeness of the historical data and may be influenced by bias or external disruptions, which should be considered when interpreting the results. Notably, potential external factors—such as the COVID-19 pandemic, advances in medical technology, and changes in healthcare access—were not incorporated into the model due to limitations in the available data, which may influence the precision of the projections. Parameter estimation and model fitting were based on the GBD 2021 dataset (22). All analyses and visualizations were conducted via R software (version 4.4.2). A *p*-value <0.05 was considered to indicate statistical significance.

### 2.6 Ethical considerations

Ethical approval was not required for this study as it was based solely on publicly available data. The study followed the Guidelines for Accurate and Transparent Health Estimates Reporting (GATHER) (23).

## 3 Results

### 3.1 Trends in the incidence, mortality, and DALYs of IBD in children and adolescents

**Figure 1:**
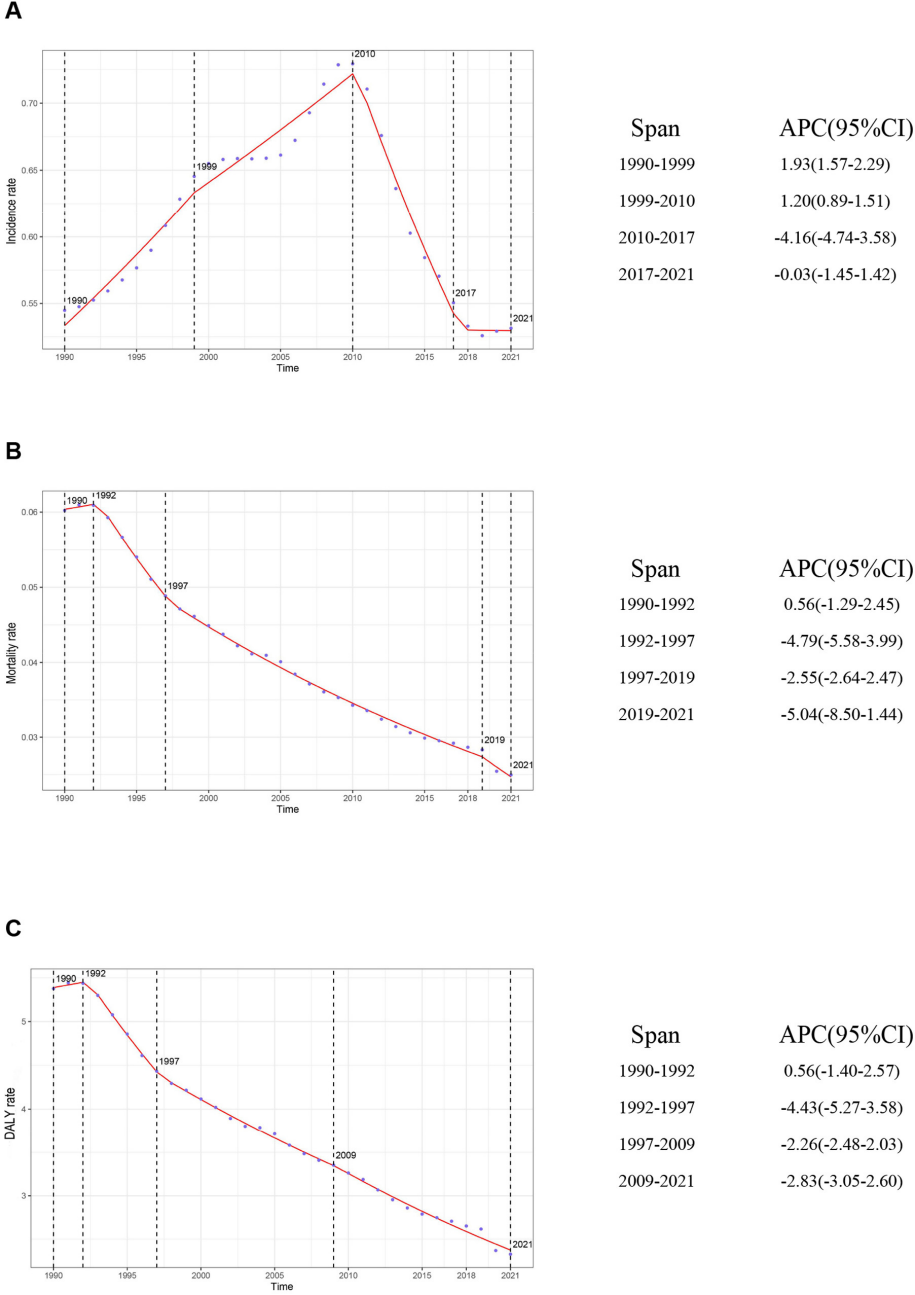
Global trends in APC and rates of IBD incidence, mortality, and DALYs in children and adolescents, 1990–2021. **(A)** Incidence rate; **(B)** Mortality rate; **(C)** DALY rate. APC, annual percentage change; IBD, inflammatory bowel disease; DALYs, disability-adjusted life years.

On the basis of data from the Global Burden of Disease Study 2021 (GBD 2021), the global burden of inflammatory bowel disease (IBD) among individuals aged 0–19 years underwent dynamic changes from 1990 to 2021. The number of incident cases increased from 12,305.32 (95% UI: 10,068.38–15,002.40) in 1990 to 14,007.76 (95% UI: 11,074.23–17,723.37) in 2021, reflecting a growth of approximately 13.8%. However, the incidence rate declined slightly, from 0.54 per 100,000 (95% UI: 0.45–0.66) in 1990 to 0.53 per 100,000 (95% UI: 0.42–0.67) in 2021, with an estimated annual percentage change (EAPC) of −0.03% (95% CI: −0.44–0.38), indicating an overall stable trend (Supplementary Table S1). Three distinct phases were observed: a period of rapid increase between 1990 and 1999 (APC: 1.93%, 95% CI: 1.57–2.29); a marked decline from 2010 to 2017 (APC: −4.16%, 95% CI: −4.74–−3.58); and a stabilization phase from 2017 to 2021 (APC: −0.03%, 95% CI: −1.45–1.42) (Figure 1A).

The number of IBD-related deaths among children and adolescents decreased significantly, from 1,360.22 (95% UI: 883.75–1,985.92) in 1990 to 657.78 (95% UI: 496.21–780.84) in 2021—a reduction of approximately 51.6%. The mortality rate decreased from 0.06 (95% UI: 0.04–0.09) to 0.02 (95% UI: 0.02–0.03), with an EAPC of −2.82 (95% CI: −2.92–−2.72) (Supplementary Table S2). The sharpest decline occurred between 1992 and 1997 (APC: −4.79%, 95% CI: −5.58–−3.99). A renewed acceleration in mortality reduction was observed from 2019 to 2021 (APC: −5.04%, 95% CI: −8.50–−1.44) (Figure 1B).

Disability-adjusted life years (DALYs)—a comprehensive measure of health loss—showed trends consistent with those of incidence and mortality. The total DALYs decreased from 121,429.41 (95% UI: 80,286.72–175,047.89) in 1990 to 61,351.82 (95% UI: 48,237.59–73,253.33) in 2021, representing a 49.5% reduction. The DALY rate declined from 5.38 (95% UI: 3.55–7.75) to 2.33 (95% UI: 1.83–2.78), with an EAPC of −2.66 (95% CI: −2.74–−2.57) (Supplementary Table S3). The most pronounced decline occurred from 1992 to 1997 (APC: −4.43%, 95% CI: −5.27–−3.58). After 2009, the rate of decline slowed (APC: −2.83%, 95% CI: −3.05–−2.60) (Figure 1C).

In 2021, substantial regional disparities in the IBD burden were evident. Australasia recorded the highest incidence rate (2.85, 95% UI: 2.26–3.64), followed by Western Europe (2.71, 95% UI: 2.16–3.46), whereas Central Latin America (0.12, 95% UI: 0.09–0.16) and East Asia (0.26, 95% UI: 0.20–0.33) had the lowest incidence rates. Regarding mortality, Western Sub-Saharan Africa showed the highest rate (0.06, 95% UI: 0.04–0.08), in contrast to the High-income Asia Pacific region and Oceania, which reported the lowest mortality (both approximately 0.00). Central Asia had the highest DALY rate (6.06, 95% UI: 4.95–7.53), followed by Western Sub-Saharan Africa (4.78, 95% UI: 2.97–6.41), whereas Oceania (0.34, 95% UI: 0.24–0.51) and the High-income Asia Pacific region (0.78, 95% UI: 0.60–1.01) had the lowest DALY rates. These findings indicate a correlation between SDI and IBD burden, reflecting substantial global heterogeneity (Supplementary Tables S1–S3). Although a general trend of higher DALY rates in lower-SDI regions was observed, this association is influenced by the differing contributions of Years of Life Lost (YLL) and Years Lived with Disability (YLD). In low-SDI settings, limited access to healthcare and delayed diagnosis may contribute to higher mortality, with YLL accounting for a larger proportion of DALYs. In contrast, high-SDI regions tend to have improved survival and long-term disease management, resulting in a relatively greater contribution from YLD.

**Figure 2:**
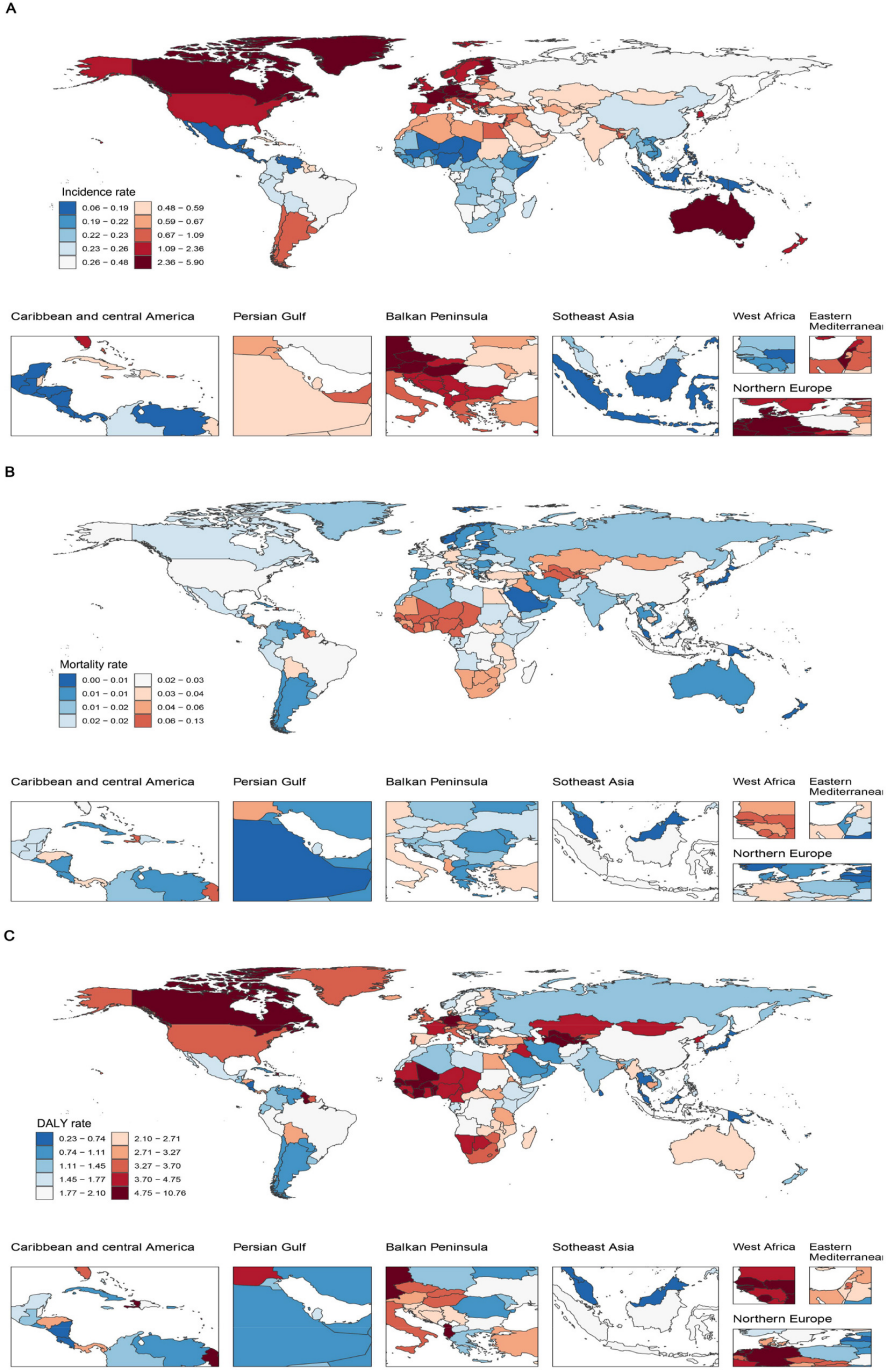
IBD burden in children and adolescents across 204 countries in 2021. **(A)** Incidence rate; **(B)** Mortality rate; **(C)** DALY rate. IBD, inflammatory bowel disease; DALYs, disability-adjusted life years.

At the national level, San Marino had the highest incidence rate (5.83, 95% UI: 4.63–7.55), whereas Mexico reported the lowest (0.06, 95% UI: 0.04–0.08). Tajikistan had the highest mortality (0.12, 95% UI: 0.07–0.22) and DALY rate (10.66, 95% UI: 5.79–19.03), whereas Singapore had the lowest mortality (0.0008, 95% UI: 0.0007–0.0009) and DALY rate (0.23, 95% UI: 0.17–0.32). These findings underscore significant global disparities in healthcare infrastructure, socioeconomic development, and medical service capacity (Figure 2).

### 3.2 SDI regional differences and frontier analysis

**Figure 3:**
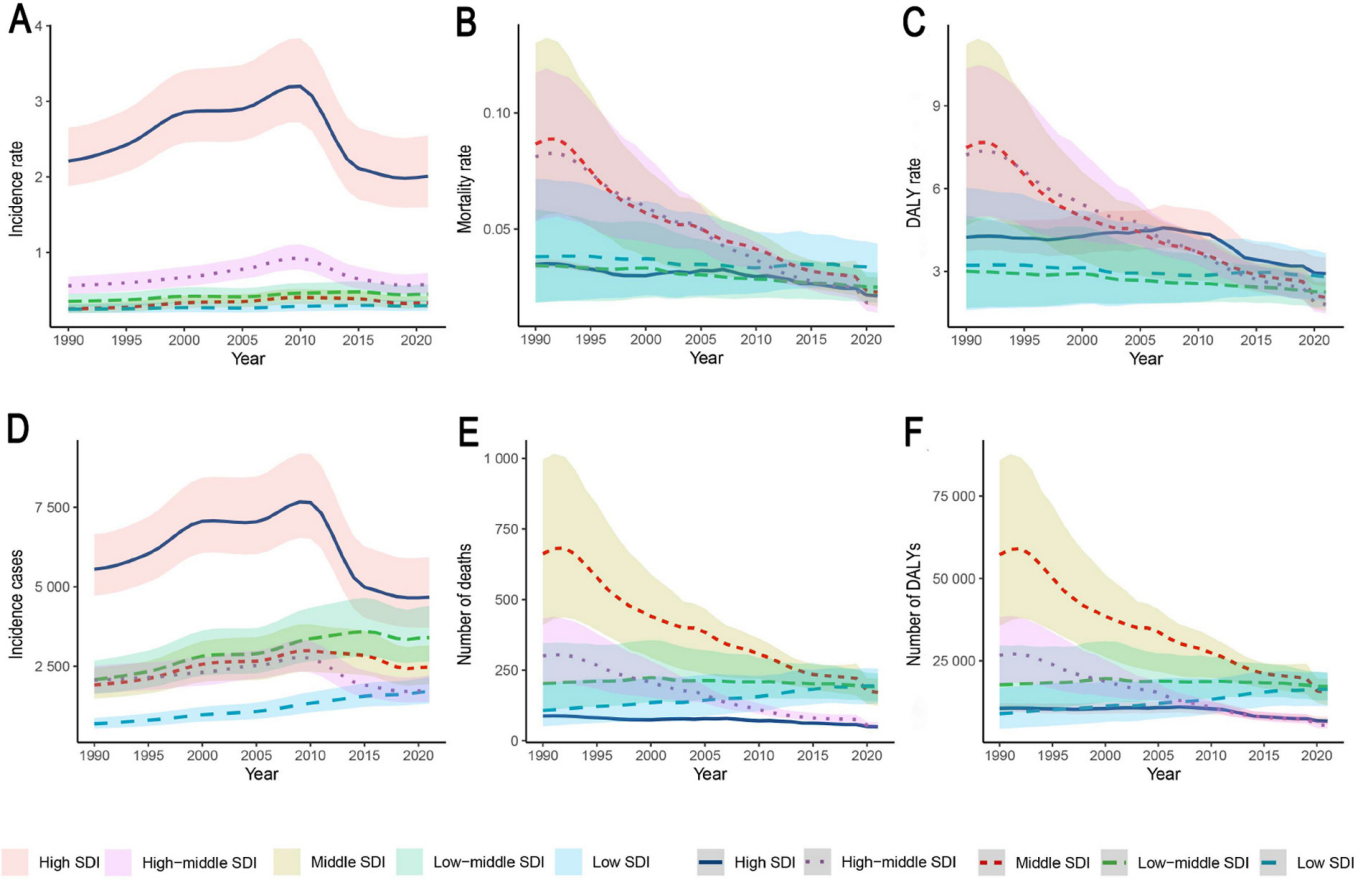
IBD burden trends in children and adolescents across five SDI regions, 1990–2021. The shaded areas represent the 95% uncertainty intervals (UIs). **(A)** Incidence rate; **(B)** Mortality rate; **(C)** DALY rate; **(D)** Incident cases; **(E)** Number of deaths; **(F)** Number of DALYs. SDI, sociodemographic Index; IBD, inflammatory bowel disease; DALYs, disability-adjusted life years.

Over the 30-year period from 1990 to 2021, the incidence of IBD among children and adolescents increased significantly in all SDI regions, except in high-SDI (EAPC: −0.45, 95% CI: −1.06–0.17) and high-middle-SDI regions (EAPC: 0.36, 95% CI: −0.28–1.00). In contrast, mortality and DALY rates declined significantly in all SDI regions. Stratified by SDI in 2021, high-SDI regions exhibited moderate incidence (2.01, 95% UI: 1.60–2.55), low mortality (0.02, 95% UI: 0.02–0.02), and DALY rates of 2.92 (95% UI: 2.45–3.50). In contrast, low-SDI regions had lower incidence (0.29, 95% UI: 0.23–0.37) but higher mortality (0.03, 95% UI: 0.02–0.04) and comparable DALY rates (2.81, 95% UI: 1.94–3.67). Notably, incidence rates increased significantly in low-SDI regions (EAPC: 0.63, 95% CI: 0.56–0.71) but declined in high-SDI regions over the same period (Supplementary Table S1–S3; Figure 3).

**Figure 4:**
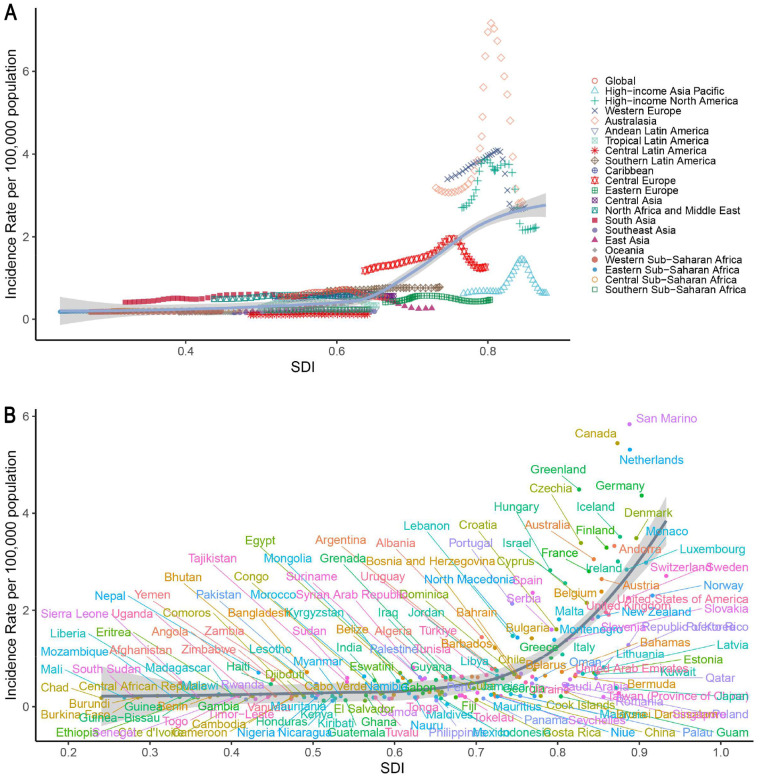
Associations between the SDI and IBD incidence rates in children and adolescents from 1990 to 2021. **(A)** Global and 21 GBD regions; **(B)** 204 countries and territories. IBD, inflammatory bowel disease; SDI, sociodemographic Index.

Specifically, in high-SDI regions such as Australasia, Western Europe, and North America continued to report relatively high incidence rates and case numbers. The incidence rate in these regions was 2.01 per 100,000 (95% UI: 1.60–2.55), with 4,670.87 new cases (95% UI: 3,718.23–5,934.98). Despite the stabilization of trends (EAPC: −2.82, 95% CI: −2.92–−2.72; *p* < 0.05), DALY rates in high-SDI regions surpassed those in lower-SDI regions after 2005. Conversely, low-SDI regions such as Central Latin America and South Asia had lower incidence rates (0.29 per 100,000, 95% UI: 0.23–0.37), but comparatively higher mortality (0.03, 95% UI: 0.02–0.04) and DALY rates (2.81, 95% UI: 1.94–3.67). Temporal patterns indicated that most high-SDI regions reached peak incidence around 2000, followed by a subsequent decline (e.g., post-2010 in Western Europe), whereas rapidly developing regions, such as East Asia, exhibited an inverted U-shaped trend from 1990 to 2021. Spearman's rank correlation coefficient indicated a strong ecological association between IBD incidence rates in individuals under 20 years and the Socio-demographic Index (SDI) across 204 countries/territories in 2021 (*ρ* = 0.7062, 95% CI: 0.6165–0.7774, *p* < 0.001), as well as across 21 GBD regions from 1990 to 2021 (*ρ* = 0.7601, 95% CI: 0.7268–0.7919, *p* < 0.001). This association was consistently observed across all GBD super-regions (*ρ* range: 0.58–0.82). Further consideration of the DALY composition discussed above facilitates a more comprehensive understanding of the regional variations in disease burden (Supplementary Table S1; Figure 4).

**Figure 5:**
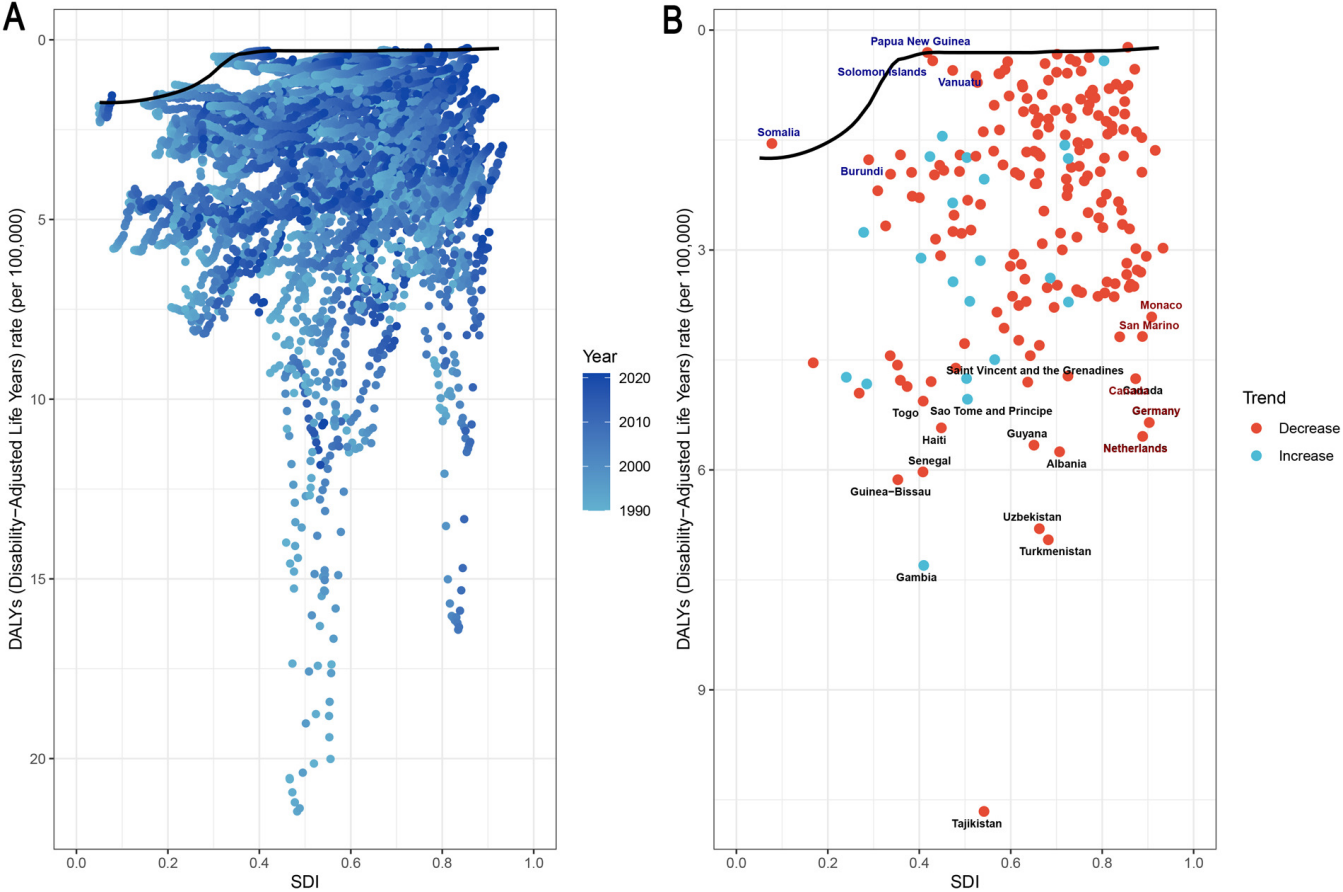
Associations between SDI and IBD DALYs in children and adolescents, 204 countries, 1990–2021. **(A)** Temporal trends; **(B)** country-level differences. Black markers: the top 15 countries with the largest effective differences; blue markers: frontier countries with low SDIs and low effective differences; red markers: countries with high SDIs but relatively high effective differences compared with their development levels. IBD, inflammatory bowel disease; DALYs, disability-adjusted life years; SDI, sociodemographic index.

Frontier analysis revealed opportunities for improving pediatric and adolescent IBD outcomes across development levels. As shown in Figure 5A, most countries with an SDI above 0.6 experienced decreasing DALY burdens over time. Figure 5B indicates that while many high-SDI countries (e.g., Germany, Canada, and the Netherlands) are approaching the frontier line, some countries with moderate-to-high SDI levels still report elevated DALY rates.

### 3.3 Age-stratified and sex-specific differences

**Figure 6:**
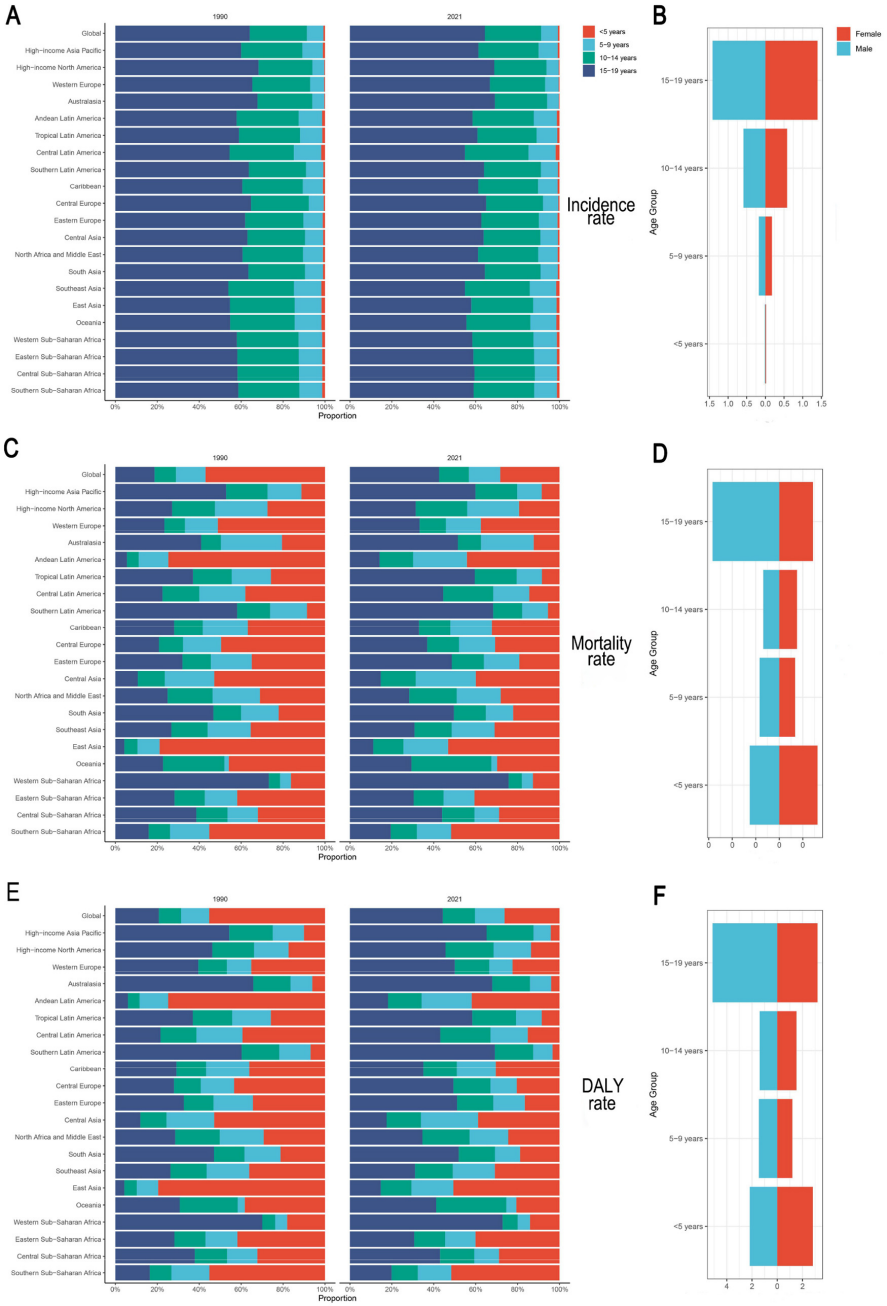
Age- and sex-specific IBD rates in children and adolescents, 1990 and 2021. **(A,B)** Incidence rate; **(C,D)** Mortality rate; **(E,F)** DALY rate. IBD, inflammatory bowel disease; DALYs, disability-adjusted life years.

We compared the IBD burden across four age groups in 1990 and 2021. The 15–19-year group consistently presented the highest incidence rate, accounting for 42.6% of all incident cases in 2021. This group also had the highest mortality and DALY rates (0.04, 95% UI: 0.03–0.05; and 4.19, 95% UI: 3.36–5.07, respectively). Although children under five years of age had the lowest incidence (0.8% of total cases), their mortality (0.03, 95% UI: 0.02–0.04) and DALY rates (2.49, 95% UI: 1.63–3.43) ranked second-highest, showing a significant increase since 1990. The 5–9 and 10–14-year groups presented relatively stable trends in all indicators (Figures 6A,C,E).

With respect to sex differences, boys had a higher IBD incidence across all age groups except those <5 years. In the 15–19 group, the incidence rate was 1.42 (95% UI: 1.10–1.85) for boys and 1.40 (95% UI: 1.08–1.84) for girls. Mortality and DALY rates were slightly higher for girls in the <5 and 10–14 groups, and for boys in the 5–9 and 15–19 groups. Notably, boys aged 15–19 years had significantly higher mortality and DALY rates than girls did, and the trend in the <5 group reversed (Figures 6B,D,F). Beyond the rates, the absolute case numbers for each indicator showed comparable patterns (Supplementary Figure S1).

We performed a sensitivity analysis of IBD incidence and disease burden trends from 1990 to 2021 across three pediatric age groups: 2–4 years, <5 years, and 5–9 years. Trends in the 2–4 and <5 groups were largely consistent, whereas the 5–9 group displayed a distinct pattern, reflecting different disease characteristics compared with very early-onset IBD. These results underscore the biological heterogeneity among pediatric age groups. Given the limitations of age stratification in the GBD database, current groupings may mask more nuanced age-specific epidemiological patterns in VEO-IBD. Nevertheless, the <5 group's overall trend remained robust, and differences observed in the 5–9 group did not alter the main conclusions regarding VEO-IBD burden. This sensitivity analysis provides a reasonable assessment of the uncertainty introduced by age grouping and highlights the need for more refined age stratification in future research to improve estimates (Supplementary Table S4).

### 3.4 Future projections

Based on GBD 2021 data and simulation results from the Bayesian age–period–cohort (BAPC) model, we projected incidence, mortality, and DALY trends of IBD among children and adolescents from 2022 to 2050. The incidence rate is expected to decline slightly from 2022 to 2023, followed by a gradual increase, potentially reaching 0.71 per 100,000 (95% UI: 0.20–1.20) by 2050—surpassing the historical peak in 2010 (0.69, 95% UI: 0.68–0.70). This trend suggests that IBD will likely continue to pose a significant health threat to young populations, particularly in upper-middle SDI countries.

**Figure 7:**
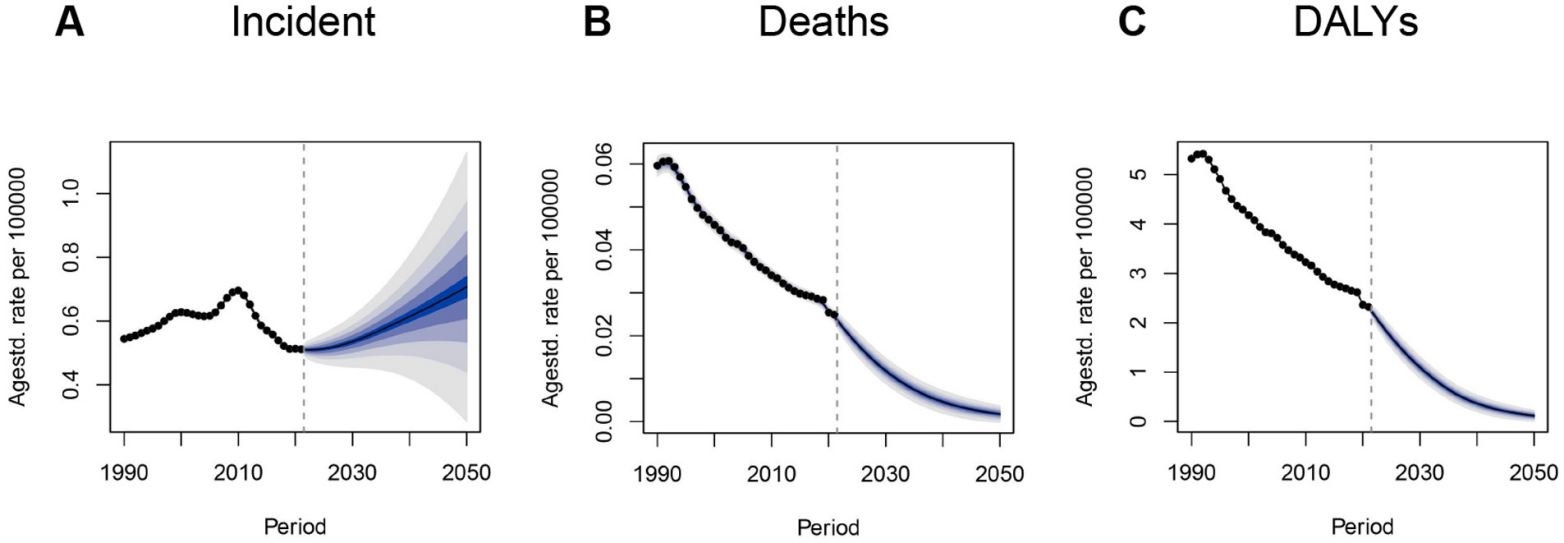
BAPC model projections of IBD incidence, mortality, and DALYs to 2050 in children and adolescents. **(A)** Incidence rate; **(B)** Mortality rate; **(C)** DALY rate. BAPC, Bayesian age–period–cohort model; IBD, inflammatory bowel disease; DALYs, disability-adjusted life years.

In contrast, IBD-related mortality and DALY rates are projected to decline steadily. The mortality rate is expected to fall below 0.01 per 100,000 by 2040, reflecting continued advancements in treatment and intervention. The DALY rate is also projected to decrease substantially, reaching 0.12 per 100,000 (95% UI: −0.02–0.25) by 2050—a notable reduction from 2021. These projections suggest that, despite a possible rise in incidence, early diagnosis, expanded use of biologics, and better disease management will likely improve overall patient outcomes (Figure 7). When analyzed across different age groups, although variations in the magnitude of predicted rates were observed, all four age cohorts exhibited similar temporal patterns (Supplementary Figure S2).

However, regional disparities may persist. In particular, low-SDI countries may see slower progress in reducing disease burden. Therefore, context-specific IBD strategies—including earlier detection, improved pediatric chronic disease management, and more equitable distribution of treatment resources—will be critical for meeting future challenges.

## 4 Discussion

### 4.1 Public health implications of pediatric IBD

Recent global trends highlight profound disparities in the pediatric IBD burden. While the incidence has stabilized or declined in high-income regions (e.g., Western Europe and North America), industrializing countries—especially East Asia (EAPC: 2.01, 95% CI: 1.02–3.19)—have experienced rapid increases. This disparity aligns with distinct epidemiological stages: an emerging phase (e.g., India, sub-Saharan Africa) characterized by limited diagnostic capacity and under-recognition of cases; an accelerating incidence phase (e.g., China, Malaysia, parts of South America) where incidence is rising alongside developing healthcare infrastructure; and a compounding prevalence phase in high-income settings (e.g., Canada, the UK, Nordic countries), where stabilized incidence, disease chronicity, and improved survival collectively drive continued increases in prevalence (24). These differences are consistent with SDI gradients: early screening programs, multidisciplinary care models, and widespread biologic use are likely associated with disease control in high-income countries, whereas low- and middle-income regions continue to struggle with diagnostic delays, limited resources, and competing public health priorities (25, 26).

Many middle- and low-income countries are facing a “double burden” of environmental exposures—struggling with both high infectious disease loads and rapid urbanization. This transition, often associated with a westernized lifestyle, has been hypothesized to contribute to of the rising incidence of immune-mediated diseases like IBD (27). Changes in diet—such as increased intake of high-fat, high-sugar, low-fiber foods and exposure to food additives (e.g., emulsifiers, preservatives)—can disrupt gut barrier function, alter microbial composition, and promote chronic inflammation (28, 29). Notably, ultra-processed foods (UPFs), which often contain high levels of such additives, have become increasingly common in middle-income countries such as Brazil, India, and South Africa, now accounting for roughly 30% of total dietary intake, with sales continuing to rise (30). In additon, Environmental pollutants, including contaminated water, particulate matter (PM2.5), and secondhand smoke exposure, may further exacerbate mucosal oxidative stress and immune dysregulation. Supporting this, a study from Hefei, China, reported a link between higher air pollutant exposure and increased IBD risk in a rapidly industrializing region, underscoring the role of environmental triggers in developing countries (31). Building on these findings, we hypothesize that the combined impact of dietary additives and environmental pollutants may act synergistically and be associated with the rising incidence of pediatric IBD in such settings, warranting targeted investigation in future research.

The “hygiene hypothesis” suggests that reduced exposure to commensal microbes in early life may impair immune regulation and increase susceptibility to IBD. Conversely, in settings with poor sanitation, frequent gastrointestinal infections may damage the mucosal barrier and activate immune pathways such as Th17/IL-23, which may in turn be linked to persistent inflammation (32–35). In India, for instance, the number of IBD cases more than doubled between 1990 and 2020 (36). Early exposure to antibiotics—particularly in unregulated settings—has also emerged as a major risk factor. Antibiotics reduce microbial diversity and impair immune tolerance, predisposing individuals to dysregulated inflammation (37, 38). Notably, early use of broad-spectrum antibiotics such as ampicillin has been shown to disrupt the gut microbiome and reduce the number of colonic RORγt^+^Foxp3^+^ regulatory T cells, thereby increasing the risk of abnormal immune responses (39).

Pediatric IBD has long-term implications beyond gastrointestinal symptoms. Recurrent hospitalizations, school disruptions, and social isolation can hinder developmental and psychological well-being. Parental caregivers often experience financial hardship, emotional stress, and anxiety (40, 41). In resource-limited settings, inadequate social safety nets may further deepen cycles of poverty and health inequity. Pediatric IBD, therefore, is not just a clinical condition—it is a pressing public health issue.

### 4.2 The implications of VEO-IBD and indirect evidence

Very early-onset IBD (VEO-IBD) presents unique challenges because of its higher likelihood of monogenic etiology, prolonged disease course, and limited research coverage (42). Our findings suggest that children under the age of five, although representing a small proportion (∼0.8%) of total IBD cases, experience disproportionately high mortality and DALY rates. Prior genetic studies have identified rare monogenic variants affecting epithelial barrier integrity, neutrophil function, and T/B-cell immunity in VEO-IBD patients (43, 44). These defects may contribute to a more complicated clinical course in patients with VEO-IBD.

However, diagnosis is difficult: symptoms in toddlers may mimic allergies or infections, leading to delays in appropriate treatment (45). Standard therapies such as corticosteroids may be ineffective in monogenic subtypes. While hematopoietic stem cell transplantation (HSCT) offers a potential cure, it involves considerable risks (46). Molecular diagnostic methods such as whole-exome sequencing can improve precision but remain costly and logistically challenging (47).

Although the <5-year-old age group was used as a proxy for VEO-IBD due to data availability, this approach has several limitations. First, the age cutoff does not perfectly align with clinical definitions of VEO-IBD, which typically include children under 6 years; thus, using the <5-year group may slightly underestimate the true burden by excluding some affected children aged 5. Second, discrepancies in age boundaries across different studies or registries may lead to inconsistencies when comparing our findings with external data. Third, this proxy may overlook distinctive clinical or genetic features of VEO-IBD, particularly in rare monogenic subtypes. Therefore, when drawing clinical inferences for VEO-IBD from our findings, caution is warranted, as the <5-year group is only an imperfect proxy for the <6-year definition and may not fully reflect its clinical and genetic characteristics.

### 4.3 Clinical and policy implications

Coordinated clinical and public health strategies are needed to reduce the burden of pediatric IBD. Early diagnosis is crucial—screening tools based on indicators such as chronic diarrhea, elevated C-reactive protein, and fecal calprotectin should be integrated into primary care workflows. For example, incorporating fecal calprotectin testing into Integrated Management of Childhood Illness (IMCI) guidelines could markedly enhance early detection in resource-limited settings—such as sub-Saharan Africa, South Asia, and Southeast Asia—where advanced diagnostic tools are often unavailable. Standardized protocols and EHR-based alerts can facilitate early identification, particularly in high-risk groups.

Routine genetic screening should be considered for suspected VEO-IBD patients to identify monogenic causes and inform personalized therapies. While some patients may benefit from HSCT, others could be candidates for clinical trials exploring microbiota-based interventions such as fecal microbiota transplantation (FMT). Comprehensive care teams—including gastroenterologists, nutritionists, psychologists, and social workers—are essential for long-term management.

At the system level, countries should develop regional pediatric IBD referral networks based on disease prevalence and service capacity. Digital health platforms, such as telemedicine and remote consultation services, facilitate shared care models, strengthen communication across institutions, and help overcome geographic and infrastructural barriers. These initiatives enhance patient access to specialist care, enable continuous disease management, and promote training and collaboration among healthcare providers—particularly in resource-limited and remote regions. Access to essential biologics (e.g., anti-TNFα or anti-IL-12/23 agents) should also be expanded through formal inclusion, biosimilar approval, and pooled procurement. Together with standardized diagnostic protocols and regional referral systems, these targeted strategies help build a sustainable and equitable global healthcare framework for pediatric IBD.

Family and school-based support systems also matter. Patient education initiatives, school accommodations, and peer networks can help reduce stigma and improve resilience.

### 4.4 Data limitations and future research directions

Although the GBD database provides a robust foundation for global comparison, it has limitations. Broad age groupings (e.g., <5 years) may mask important heterogeneity—infantile-onset IBD (<2 years) is usually monogenic, whereas preschool-onset IBD (3–6 years) may involve gene–environment interactions. More granular clinical registries, such as the ESPGHAN and EUROKIDS databases, which capture detailed patient-level information, could enhance sensitivity in future validations; however, these data were not available for this study and represent a crucial area for future research.

It should also be noted that, as the GBD 2021 dataset ends in 2021, our estimates may not fully capture post-pandemic healthcare disruptions, such as diagnostic delays and treatment interruptions, which could influence recent incidence patterns. In addition, the lack of stratification by IBD subtypes (Crohn's disease, ulcerative colitis, and IBD-unclassified) in the GBD dataset limits our ability to examine potentially divergent temporal trends between these subtypes, which have been reported in regional registry studies.

Reporting bias is another consideration: high-income countries have reliable registries, whereas low-income countries may rely on fragmented hospital records, potentially underestimating the disease burden (48). GBD projections depend on the completeness of historical data and do not incorporate risk factor trends (49). Moreover, they have not been externally validated against independent regional registry data, which should be considered when interpreting the forecasted trends. Finally, relying on national-level Socio-demographic Index (SDI) data may mask significant within-country disparities, such as urban-rural differences and intersectional vulnerabilities faced by populations like migrant children. Future research should incorporate subnational SDI stratification or integrate poverty mapping to more accurately capture these critical dimensions of health equity.

Future research priorities include the following:

(1) establishing high-quality, globally harmonized pediatric IBD registries;
(2) applying AI to EHR data for early prediction and classification;
(3) investigating long-term outcomes including cardiovascular, neurodevelopmental, and metabolic complications (50, 51).

## 5 Conclusion

This study provides a comprehensive analysis of the global burden of inflammatory bowel disease among children and adolescents aged 0–19 years from 1990 to 2021. While the incidence has remained relatively stable, regional disparities have been pronounced—high-income regions have shown plateauing trends, whereas low- and middle-income countries have experienced rising burdens. Although VEO-IBD accounted for a small proportion of cases, it was associated with disproportionately high mortality and DALYs, highlighting the need for improved early recognition. Despite reductions in mortality and DALY rates globally, the burden remains substantial in low-SDI areas. To address this, we recommend expanding early screening, strengthening referral networks, and improving resource distribution. Future efforts should prioritize building integrated registries and advancing AI-driven diagnostics to reduce long-term health burdens in pediatric IBD patients.

## Data Availability

The datasets analyzed in this study are publicly available and were obtained from the GBD 2021 study via the Global Health Data Exchange (GHDx): https://ghdx.healthdata.org/gbd-2021. The data sheets uploaded as Supplementary Material represent only the specific subsets used for analysis in this study.
