# Supplementary material for: Global burden of inflammatory bowel disease in children and adolescents, 1990–2021: trends, age-specific patterns and future projections
Source: Front Pediatr. 2025 Sep 3;13:1670440. doi: 10.3389/fped.2025.1670440 (PMC12440882; doi:10.3389/fped.2025.1670440)
Supplement: Supplementary file 1 [file Supplementaryfile1.docx]

Supplementary Material

**Table S1.** Global and regional IBD incidence trends in children and adolescents, 1990–2021.

**Table S2.** Global and regional IBD mortality trends in children and adolescents, 1990–2021.

**Table S3.** Global and regional IBD DALYs trends in children and adolescents, 1990–2021.

**Table S4.** Total percentage change in incidence, deaths, and DALYs of IBD across age groups (2–4 years, <5 years, and 5–9 years) from 1990 to 2021, global estimates.

**Table S5.** Dataset containing incidence, deaths, and DALYs of IBD in children and adolescents in 204 countries and territories from 1990 to 2021.

**Table S6.** Dataset containing incidence, deaths, and DALYs of IBD in children and adolescents in the global total, five SDI regions, and 21 GBD regions from 1990 to 2021.

**Figure S1.** Age- and sex-specific IBD numbers in children and adolescents, 1990 and 2021.

**Figure S2.** BAPC model projections of IBD incidence, mortality, and DALYs to 2050 in children and adolescents of 4 age groups.

**Table S1.** Global and regional IBD incidence trends in children and adolescents, 1990–2021

| **Location** | **1990** | | **2021** | | **1990-2021** |
| --- | --- | --- | --- | --- | --- |
|  | incident cases  (95% UI) | incident rate  per 100,000(95% UI) | incident cases  (95% UI) | incident rate  per 100,000(95% UI) | EAPC  (95% CI) |
| Global | 12305.32(10068.38,15002.40) | 0.54(0.45,0.66) | 14007.76(11074.23,17723.37) | 0.53(0.42,0.67) | -0.03(-0.44,0.38) |
| **SDI** | | | | | |
| High SDI | 5553.05(4719.74,6659.50) | 2.21(1.88,2.65) | 4670.87(3718.23,5934.98) | 2.01(1.60,2.55) | -0.45(-1.06,0.17) |
| High-middle SDI | 2059.82(1657.22,2517.98) | 0.56(0.45,0.68) | 1735.47(1377.39,2210.13) | 0.57(0.45,0.73) | 0.36(-0.28,1.00) |
| Middle SDI | 1911.35(1486.43,2458.88) | 0.25(0.19,0.32) | 2473.71(1929.90,3156.37) | 0.33(0.26,0.42) | 1.17(0.76,1.58) |
| Low-middle SDI | 2077.49(1627.22,2671.90) | 0.35(0.28,0.45) | 3403.43(2647.17,4396.25) | 0.45(0.35,0.58) | 0.91(0.74,1.08) |
| Low SDI | 689.49(537.65,871.12) | 0.25(0.19,0.31) | 1712.19(1332.33,2159.50) | 0.29(0.23,0.37) | 0.63(0.56,0.71) |
| **Regions** | | | | | |
| Andean Latin America | 42.15(32.41,54.19) | 0.22(0.17,0.29) | 58.84(45.48,75.76) | 0.25(0.19,0.32) | 0.46(0.36,0.57) |
| Australasia | 199.73(160.78,263.16) | 3.18(2.56,4.20) | 214.74(170.26,274.15) | 2.85(2.26,3.64) | 1.32(0.12,2.52) |
| Caribbean | 75.11(59.04,94.59) | 0.50(0.39,0.63) | 80.97(62.74,101.87) | 0.53(0.41,0.67) | 0.33(0.20,0.45) |
| Central Asia | 162.39(126.88,202.55) | 0.51(0.40,0.64) | 194.79(153.88,248.23) | 0.56(0.44,0.72) | 0.33(0.03,0.63) |
| Central Europe | 459.41(373.54,568.45) | 1.17(0.95,1.45) | 296.59(237.93,373.30) | 1.26(1.01,1.58) | 0.43(-0.16,1.03) |
| Central Latin America | 92.84(70.79,124.01) | 0.11(0.09,0.15) | 102.05(77.26,135.29) | 0.12(0.09,0.16) | 0.36(0.20,0.52) |
| Central Sub-Saharan Africa | 59.60(46.33,74.83) | 0.19(0.15,0.24) | 173.41(135.25,220.09) | 0.24(0.18,0.30) | 0.59(0.53,0.66) |
| East Asia | 866.83(675.14,1117.08) | 0.19(0.15,0.24) | 904.38(696.57,1148.95) | 0.26(0.20,0.33) | 2.10(1.02,3.19) |
| Eastern Europe | 276.69(216.39,353.32) | 0.41(0.32,0.53) | 215.75(168.58,276.54) | 0.47(0.37,0.60) | -0.13(-0.54,0.28) |
| Eastern Sub-Saharan Africa | 197.81(153.98,249.28) | 0.18(0.14,0.22) | 501.51(387.44,632.48) | 0.22(0.17,0.28) | 0.67(0.60,0.74) |
| High-income Asia Pacific | 311.19(249.94,384.07) | 0.62(0.50,0.76) | 192.81(152.55,244.39) | 0.63(0.50,0.79) | 1.12(-0.01,2.26) |
| High-income North America | 2213.26(1873.23,2645.51) | 2.71(2.29,3.24) | 1999.77(1585.90,2548.06) | 2.23(1.77,2.85) | -1.07(-1.82,-0.32) |
| North Africa and Middle East | 863.84(681.95,1103.54) | 0.49(0.39,0.62) | 1431.15(1116.89,1809.71) | 0.61(0.47,0.77) | 0.55(0.38,0.71) |
| Oceania | 4.78(3.65,6.32) | 0.14(0.11,0.19) | 9.88(7.68,12.93) | 0.15(0.12,0.20) | 0.29(0.23,0.35) |
| South Asia | 2218.34(1714.60,2866.37) | 0.41(0.32,0.53) | 3744.64(2904.25,4878.51) | 0.55(0.42,0.71) | 1.21(0.97,1.45) |
| Southeast Asia | 347.90(270.93,451.73) | 0.16(0.12,0.21) | 424.18(329.42,544.82) | 0.19(0.14,0.24) | 0.56(0.47,0.64) |
| Southern Latin America | 130.97(101.25,170.71) | 0.68(0.52,0.88) | 151.42(118.26,194.15) | 0.78(0.61,1.00) | 0.41(0.35,0.46) |
| Southern Sub-Saharan Africa | 58.17(45.45,73.59) | 0.22(0.17,0.28) | 75.19(58.96,94.31) | 0.24(0.19,0.30) | 0.17(0.08,0.26) |
| Tropical Latin America | 196.46(153.07,249.05) | 0.28(0.22,0.36) | 221.10(170.92,285.26) | 0.33(0.26,0.43) | 0.12(-0.52,0.77) |
| Western Europe | 3339.14(2838.92,4010.96) | 3.40(2.89,4.08) | 2486.82(1977.33,3174.18) | 2.71(2.16,3.46) | -1.00(-1.50,-0.51) |
| Western Sub-Saharan Africa | 188.71(146.71,235.98) | 0.18(0.14,0.22) | 527.78(413.82,660.08) | 0.20(0.15,0.25) | 0.20(0.13,0.28) |

Abbreviations: IBD, Inflammatory bowel disease; SDI, sociodemographic Index; EAPC, estimated annual percentage change; UI, uncertainty interval; CI, confidence interval.

**Table S2.** Global and regional IBD mortality trends in children and adolescents, 1990–2021

| **Location** | **1990** | | **2021** | | **1990-2021** |
| --- | --- | --- | --- | --- | --- |
|  | number of deaths  (95% UI) | mortality rate  per 100,000(95% UI) | number of deaths  (95% UI) | mortality rate  per 100,000(95% UI) | EAPC  (95% CI) |
| Global | 1360.22(883.75,1985.92) | 0.06(0.04,0.09) | 657.78(496.21,780.84) | 0.02(0.02,0.03) | -2.82(-2.92,-2.72) |
| **SDI** | | | | | |
| High SDI | 87.28(82.25,92.62) | 0.03(0.03,0.04) | 49.37(46.74,51.91) | 0.02(0.02,0.02) | -1.21(-1.46,-0.97) |
| High-middle SDI | 300.36(197.64,433.76) | 0.08(0.05,0.12) | 52.99(42.42,65.51) | 0.02(0.01,0.02) | -4.77(-5.02,-4.51) |
| Middle SDI | 662.40(410.54,995.56) | 0.09(0.05,0.13) | 170.71(124.62,219.37) | 0.02(0.02,0.03) | -4.07(-4.25,-3.89) |
| Low-middle SDI | 202.84(106.91,345.14) | 0.03(0.02,0.06) | 190.53(139.28,238.49) | 0.02(0.02,0.03) | -1.08(-1.15,-1.02) |
| Low SDI | 106.60(51.43,200.20) | 0.04(0.02,0.07) | 193.76(132.14,255.57) | 0.03(0.02,0.04) | -0.48(-0.57,-0.38) |
| **Regions** | | | | | |
| Andean Latin America | 21.36(11.48,35.41) | 0.11(0.06,0.19) | 5.17(3.33,7.84) | 0.02(0.01,0.03) | -5.33(-5.88,-4.78) |
| Australasia | 0.55(0.49,0.61) | 0.01(0.01,0.01) | 0.68(0.60,0.76) | 0.01(0.01,0.01) | 1.28(0.34,2.24) |
| Caribbean | 7.77(4.53,13.85) | 0.05(0.03,0.09) | 5.20(2.61,9.63) | 0.03(0.02,0.06) | -1.33(-1.59,-1.08) |
| Central Asia | 41.21(25.92,52.62) | 0.13(0.08,0.17) | 23.79(19.20,29.82) | 0.07(0.06,0.09) | -2.00(-2.39,-1.60) |
| Central Europe | 17.98(15.70,20.44) | 0.05(0.04,0.05) | 3.86(3.32,4.52) | 0.02(0.01,0.02) | -2.68(-2.94,-2.42) |
| Central Latin America | 28.69(25.23,31.81) | 0.03(0.03,0.04) | 15.84(13.58,18.51) | 0.02(0.02,0.02) | -1.05(-1.47,-0.63) |
| Central Sub-Saharan Africa | 8.43(3.45,15.50) | 0.03(0.01,0.05) | 16.41(9.06,27.95) | 0.02(0.01,0.04) | -0.33(-0.47,-0.19) |
| East Asia | 734.56(431.22,1127.84) | 0.16(0.09,0.25) | 78.44(49.81,110.39) | 0.02(0.01,0.03) | -5.57(-6.06,-5.08) |
| Eastern Europe | 25.30(21.73,28.48) | 0.04(0.03,0.04) | 6.25(5.77,6.75) | 0.01(0.01,0.01) | -3.45(-3.90,-3.00) |
| Eastern Sub-Saharan Africa | 30.22(11.03,61.94) | 0.03(0.01,0.06) | 55.43(35.51,78.91) | 0.02(0.02,0.03) | -0.07(-0.17,0.02) |
| High-income Asia Pacific | 11.15(8.08,14.94) | 0.02(0.02,0.03) | 1.32(1.11,1.75) | 0.00(0.00,0.01) | -5.39(-5.82,-4.95) |
| High-income North America | 24.95(24.34,25.58) | 0.03(0.03,0.03) | 25.65(24.11,27.35) | 0.03(0.03,0.03) | -0.16(-0.46,0.14) |
| North Africa and Middle East | 61.70(28.55,125.24) | 0.03(0.02,0.07) | 53.95(39.00,76.24) | 0.02(0.02,0.03) | -1.21(-1.35,-1.07) |
| Oceania | 0.20(0.11,0.42) | 0.01(0.00,0.01) | 0.18(0.12,0.30) | 0.00(0.00,0.00) | -3.29(-3.77,-2.81) |
| South Asia | 136.28(53.64,263.28) | 0.03(0.01,0.05) | 104.30(60.58,157.10) | 0.02(0.01,0.02) | -1.75(-1.99,-1.50) |
| Southeast Asia | 63.68(34.93,105.56) | 0.03(0.02,0.05) | 44.75(27.35,56.99) | 0.02(0.01,0.02) | -1.44(-1.55,-1.33) |
| Southern Latin America | 2.83(2.60,3.06) | 0.01(0.01,0.02) | 1.41(1.27,1.58) | 0.01(0.01,0.01) | -1.66(-1.92,-1.40) |
| Southern Sub-Saharan Africa | 12.44(7.60,22.01) | 0.05(0.03,0.08) | 12.83(9.10,17.95) | 0.04(0.03,0.06) | -0.13(-0.48,0.23) |
| Tropical Latin America | 19.78(17.90,21.80) | 0.03(0.03,0.03) | 15.86(14.26,17.58) | 0.02(0.02,0.03) | 0.25(-0.07,0.56) |
| Western Europe | 45.39(43.01,47.22) | 0.05(0.04,0.05) | 23.12(21.40,24.62) | 0.03(0.02,0.03) | -1.31(-1.70,-0.93) |
| Western Sub-Saharan Africa | 65.75(48.08,89.17) | 0.06(0.04,0.08) | 163.35(98.61,220.97) | 0.06(0.04,0.08) | -0.06(-0.18,0.05) |

Abbreviations: IBD, Inflammatory bowel disease; SDI, sociodemographic Index; EAPC, estimated annual percentage change; UI, uncertainty interval; CI, confidence interval.

**Table S3.** Global and regional IBD DALYs trends in children and adolescents, 1990–2021

| **Location** | **1990** | | **2021** | | **1990-2021** |
| --- | --- | --- | --- | --- | --- |
|  | number of DALYs  (95% UI) | DALY rate  per 100,000(95% UI) | number of DALYs  (95% UI) | DALY rate  per 100,000(95% UI) | EAPC  (95% CI) |
| Global | 121429.41(80286.72,175047.89) | 5.38(3.55,7.75) | 61351.82(48237.59,73253.33) | 2.33(1.83,2.78) | -2.66(-2.74,-2.57) |
| **SDI** | | | | | |
| High SDI | 10639.90(9348.21,12226.73) | 4.23(3.72,4.87) | 6798.37(5694.08,8135.75) | 2.92(2.45,3.50) | -1.02(-1.39,-0.65) |
| High-middle SDI | 26733.59(17869.09,38322.03) | 7.22(4.83,10.35) | 5467.24(4486.80,6711.04) | 1.80(1.48,2.21) | -4.30(-4.55,-4.05) |
| Middle SDI | 57219.28(35613.44,85833.76) | 7.48(4.66,11.23) | 15423.79(11696.77,19565.19) | 2.06(1.56,2.61) | -3.91(-4.09,-3.74) |
| Low-middle SDI | 17766.87(9853.46,29720.98) | 3.01(1.67,5.03) | 17223.70(13100.52,21461.24) | 2.25(1.71,2.81) | -0.94(-1.00,-0.88) |
| Low SDI | 8998.56(4460.98,16847.42) | 3.22(1.60,6.03) | 16396.35(11318.64,21422.27) | 2.81(1.94,3.67) | -0.44(-0.53,-0.36) |
| **Regions** | | | | | |
| Andean Latin America | 1851.95(996.90,3065.05) | 9.77(5.26,16.17) | 470.35(316.50,697.58) | 1.99(1.34,2.95) | -5.19(-5.73,-4.64) |
| Australasia | 168.39(111.91,246.07) | 2.68(1.78,3.92) | 180.33(124.40,249.04) | 2.39(1.65,3.30) | 1.19(0.18,2.22) |
| Caribbean | 687.50(421.74,1208.99) | 4.55(2.79,8.01) | 476.97(261.36,847.33) | 3.13(1.71,5.55) | -1.19(-1.42,-0.96) |
| Central Asia | 3569.46(2283.49,4555.69) | 11.30(7.23,14.43) | 2097.22(1713.08,2607.47) | 6.06(4.95,7.53) | -1.91(-2.28,-1.54) |
| Central Europe | 1794.54(1562.96,2032.10) | 4.57(3.98,5.17) | 507.70(421.61,618.96) | 2.16(1.79,2.63) | -1.94(-2.23,-1.64) |
| Central Latin America | 2413.04(2113.55,2679.64) | 2.92(2.56,3.24) | 1306.08(1116.56,1517.84) | 1.53(1.31,1.78) | -1.13(-1.53,-0.73) |
| Central Sub-Saharan Africa | 729.03(312.85,1327.19) | 2.35(1.01,4.28) | 1423.47(820.40,2404.70) | 1.94(1.12,3.27) | -0.33(-0.47,-0.20) |
| East Asia | 63263.60(37049.14,97112.50) | 13.75(8.05,21.10) | 7103.76(4711.55,9874.44) | 2.06(1.37,2.86) | -5.38(-5.88,-4.89) |
| Eastern Europe | 2229.37(1916.57,2499.08) | 3.31(2.85,3.71) | 630.15(563.04,704.53) | 1.37(1.22,1.53) | -3.02(-3.39,-2.66) |
| Eastern Sub-Saharan Africa | 2631.10(1007.63,5324.65) | 2.37(0.91,4.80) | 4860.00(3234.19,6942.05) | 2.14(1.42,3.05) | -0.05(-0.15,0.04) |
| High-income Asia Pacific | 1088.73(844.79,1399.27) | 2.16(1.68,2.78) | 240.45(183.99,312.37) | 0.78(0.60,1.01) | -2.65(-3.07,-2.23) |
| High-income North America | 3388.95(2909.09,3979.00) | 4.15(3.56,4.87) | 3210.58(2760.77,3744.38) | 3.58(3.08,4.18) | -0.64(-1.15,-0.13) |
| North Africa and Middle East | 5628.09(2869.75,10961.54) | 3.18(1.62,6.20) | 5324.03(4046.59,7057.91) | 2.25(1.71,2.98) | -0.98(-1.13,-0.84) |
| Oceania | 20.62(12.46,39.74) | 0.61(0.37,1.18) | 21.86(15.28,32.52) | 0.34(0.24,0.51) | -2.57(-2.95,-2.18) |
| South Asia | 12316.47(5609.37,22559.39) | 2.27(1.03,4.16) | 10660.78(7179.90,14795.90) | 1.56(1.05,2.16) | -1.27(-1.48,-1.06) |
| Southeast Asia | 5435.73(3077.98,8998.84) | 2.47(1.40,4.09) | 3893.21(2501.16,4876.64) | 1.70(1.09,2.13) | -1.35(-1.45,-1.26) |
| Southern Latin America | 307.29(268.17,349.75) | 1.59(1.38,1.80) | 209.44(171.79,258.55) | 1.07(0.88,1.33) | -0.92(-1.07,-0.77) |
| Southern Sub-Saharan Africa | 1080.17(658.95,1914.66) | 4.08(2.49,7.24) | 1112.89(790.30,1554.70) | 3.56(2.53,4.97) | -0.13(-0.49,0.23) |
| Tropical Latin America | 1713.75(1534.99,1898.03) | 2.47(2.22,2.74) | 1364.11(1228.82,1507.78) | 2.05(1.85,2.26) | 0.11(-0.16,0.37) |
| Western Europe | 5894.55(5129.21,6822.86) | 5.99(5.22,6.94) | 3418.65(2827.26,4163.59) | 3.73(3.08,4.54) | -1.28(-1.65,-0.90) |
| Western Sub-Saharan Africa | 5217.10(3795.88,7213.33) | 4.85(3.53,6.71) | 12839.79(7966.85,17224.50) | 4.78(2.97,6.41) | -0.08(-0.19,0.04) |

Abbreviations: IBD, Inflammatory bowel disease; DALYs, disability-adjusted life years; SDI, sociodemographic Index; EAPC, estimated annual percentage change; UI, uncertainty interval; CI, confidence interval.

**Table S4.** Total percentage change in incidence, deaths, and DALYs of IBD across age groups (2–4 years, <5 years, and 5–9 years) from 1990 to 2021, global estimates.

| **Location** | **Measure** | **Metric** | **Age** | **Year Range** | **Total Percentage Change** | **95%UI** |
| --- | --- | --- | --- | --- | --- | --- |
| Global | Incidence | Rate | 2-4 years | 1990-2021 | 0.01 | (-0.01,0.03) |
|  | Incidence | Rate | <5 years | 1990-2021 | 0.04 | (0.02,0.07) |
|  | Incidence | Rate | 5-9 years | 1990-2021 | -0.04 | (-0.07,-0.02) |
|  | Deaths | Rate | 2-4 years | 1990-2021 | -0.79 | (-0.86,-0.62) |
|  | Deaths | Rate | <5 years | 1990-2021 | -0.78 | (-0.86,-0.6) |
|  | Deaths | Rate | 5-9 years | 1990-2021 | -0.53 | (-0.68,-0.29) |
|  | DALYs | Rate | 2-4 years | 1990-2021 | -0.79 | (-0.87,-0.62) |
|  | DALYs | Rate | <5 years | 1990-2021 | -0.78 | (-0.86,-0.6) |
|  | DALYs | Rate | 5-9 years | 1990-2021 | -0.52 | (-0.66,-0.28) |


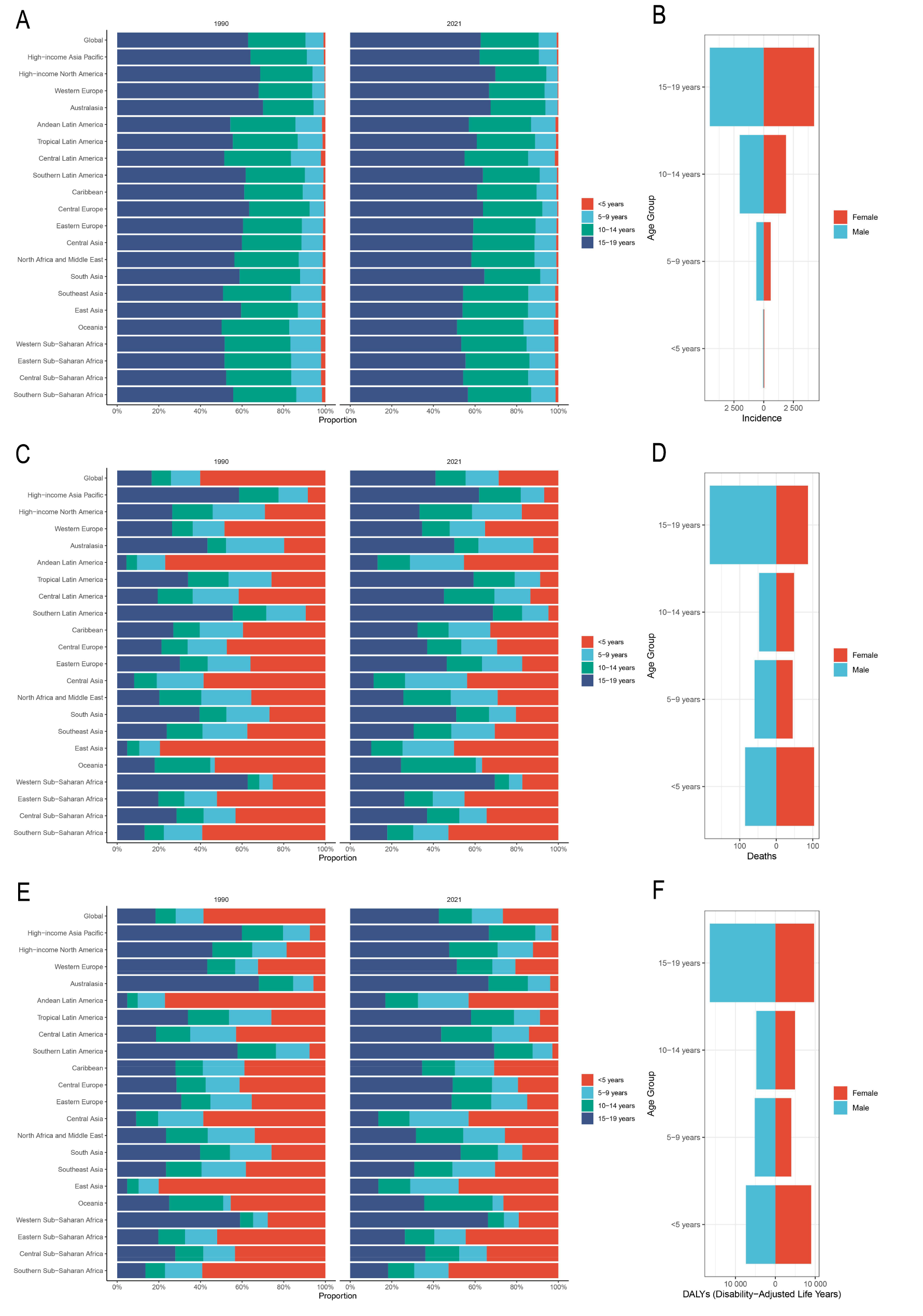


**Figure S1.** Age- and sex-specific IBD numbers in children and adolescents, 1990 and 2021. **(A)** & **(B)** Incidence number; **(C)** & **(D)** Number of deaths; **(E)** & **(F)** Number of DALYs. Abbreviations: IBD, inflammatory bowel disease; DALYs, disability-adjusted life years.

**
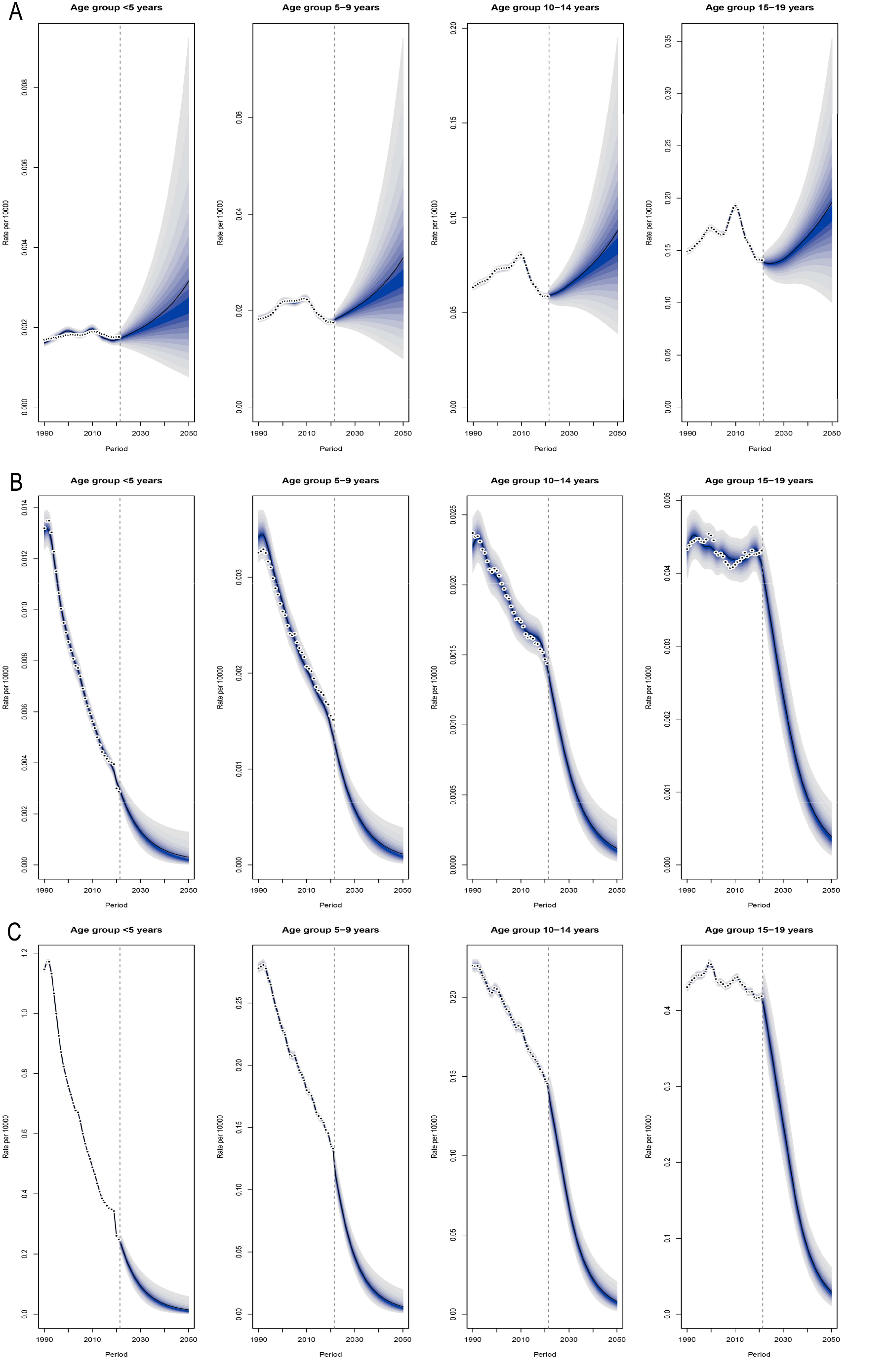
**

**Figure S2.** BAPC model projections of IBD incidence, mortality, and DALYs to 2050 in children and adolescents of 4 age groups. **(A)** Incidence rates; **(B)** Mortality rates; **(C)** DALY rates. Abbreviations: BAPC, Bayesian age–period–cohort model; IBD, inflammatory bowel disease; DALYs, disability-adjusted life years.
